# Influence of Temperature and Photoperiod on Survival and Development of *Eoreuma loftini* (Lepidoptera: Crambidae)

**DOI:** 10.3390/insects15120915

**Published:** 2024-11-23

**Authors:** James M. Villegas, Rodrigo Diaz, Michael J. Stout, Fin Papitchaya, Blake E. Wilson

**Affiliations:** 1 Dean Lee Research and Extension Center, LSU Agricultural Center, Alexandria, LA 71302, USA; 2 Department of Entomology, LSU Agricultural Center, Baton Rouge, LA 70803, USA

**Keywords:** cold tolerance, invasive species, Mexican rice borer, range expansion, degree days

## Abstract

The Mexican rice borer, *Eoreuma loftini* (Lepidoptera: Crambidae) is an economically important pest of sugarcane, rice, and corn in Louisiana, Texas, and Mexico. This pest is considered invasive in the US and is expanding its range northward. Due to its subtropical origin, *E. loftini*’s northern distribution might be limited by cold tolerance. A series of assays determined the influence of temperature and photoperiod on *E. loftini* life table parameters. Adult oviposition was reduced at temperatures > 30 °C. Egg viability was reduced at 20 and 36 °C relative to intermediate temperatures. Egg development time was greatest at 18 °C. Larvae did not develop to the pupal stage at temperatures ≤ 20 °C. Larval duration decreased with increasing temperature from 22 to 36 °C. Adult longevity decreased with increasing temperature from 18 °C (15 d) to 34 °C (7 d). Exposure to a short-day photoperiod decreased larval mortality at −5 °C. Larval survival of >80% up to 5 days at −5 °C suggests *E. loftini* cold tolerance is sufficient to minimize the influence of hard freezes on overwintering populations. Larval survival for 5 d at 40 °C was 75%. Exposure to 45 °C for periods > 1 d caused 100% mortality. These results suggest that *E. loftini* exhibits considerable thermal plasticity and cold tolerance, which may facilitate its range expansion in the United States.

## Simple Summary

The Mexican rice borer, *Eoreuma loftini* Dyar (Lepidoptera: Crambidae) is an economically important pest of sugarcane, rice, and corn in Louisiana, Texas, and Mexico. Due to its tropical and subtropical origin, *E. loftini*’s northern distribution might be limited by cold tolerance. Thus, a series of biological assays was conducted to determine the influence of temperature regimes and photoperiod on *E. loftini* life table parameters. Development assays revealed that larvae did not complete development to adulthood at temperatures < 22 °C. Adult longevity decreased with increasing temperature. Thermal tolerance assays determined that larvae have high rates of survival at freezing temperatures (−5 °C) for five days. All larvae died after one day at 45 °C. Exposure to a short-day photoperiod improved survival at freezing temperatures, but photoperiod did not influence mortality at high temperatures. These results suggest that *E. loftini* can tolerate a wide range of temperatures, which may facilitate its range expansion in the United States, although extreme high temperatures could potentially limit its spread in some areas.

## 1. Introduction

The Mexican rice borer, *Eoreuma loftini* Dyar (Lepidoptera: Crambidae), is an invasive insect pest of sugarcane, rice, corn, and other graminaceous crops endemic to Mexico [1,2]. The species has been expanding its range northward and eastward along the coast of the Gulf of Mexico since 1980, reaching Louisiana in 2008 [3]. The species has continued its northward and eastward range expansion in Louisiana, now infesting most of the state’s sugarcane and rice acreage [4]. Further, separate introductions were detected in Florida in 2012 [5] and Georgia in 2021 [6]. The Florida introduction was detected in forested habitat of Levy County and expansion towards crop production regions has been relatively slow [5]. The Georgia detection occurred in non-commercial sugarcane fields in Effingham County, representing the northernmost detection to date [6]. This species has also been expanding its range northward in California [7]. This expansion has raised concerns about the species’ ability to become established in the corn- and rice-producing regions of the Mississippi Delta and broader southeastern US.

The potential distribution of *E. loftini* in the US is likely to be limited to the North by winter temperatures and the subtropical species’ cold tolerance. Indeed, winter mortality is a key factor limiting the northward establishment of tropical and subtropical insect invaders [8]. *Eoreuma loftini* has remained established in central Louisiana despite winter temperatures regularly dropping below those that caused 100% mortality of *E. loftini* larvae in previous studies [9]. This suggests that populations of this species may have evolved greater cold tolerance during its northward expansion over the past >40 years. Changes in *E. loftini* biology may have also altered the influence of temperature and photoperiod on larval development and adult reproductive parameters. Thus, the objectives of this study are to (1) determine the influence of temperature on *E. loftini* development and reproductive parameters, (2) examine the effect of photoperiod on *E. loftini* biology, and (3) assess *E. loftini* survival under extreme temperatures and varied photoperiods.

## 2. Materials and Methods

### 2.1. Insect Source

The *E. loftini* utilized in this study were from a colony maintained continuously in the laboratory following the protocol outlined by Villegas et al. (2021) [10]. This laboratory colony originated from *E. loftini* larvae collected from rice plants in Acadia Parish, Louisiana from 2018 to 2020. Larvae were reared on a multiple species artificial diet formulated for Lepidoptera (Southland Products Inc., Lake Village, AR, USA) in 30 mL clear portion containers (Conex^®^ Complements™, Dart Container Corporation, Mason, MI, USA) until pupation. Pupae were collected and sexed according to Butts and Canto [11] and equal numbers of male and female pupae were then placed in 3 L plastic containers (Prolon^®^, Cambro Manufacturing Co., Huntington Beach, CA, USA) with a cheese cloth cover for ventilation and with germination paper rolled into cylinders (Anchor Seed Solutions, Anchor Paper Co., St. Paul, MN, USA) serving as substrate for oviposition. Upon eclosion, adult moths were provided with a 1:1 mixture of honey and beer along with distilled water as their food source. Eggs were collected and allowed to hatch in 8-cell plastic trays (BIO-SMRT-8, C-D International, Allentown, NJ, USA), with each cell containing a moistened cotton ball. The colony was maintained under controlled conditions at 28 °C with 60 ± 10% RH and a photoperiod of 14:10 h (L:D).

### 2.2. Eoreuma loftini Development Under Varying Temperature

#### 2.2.1. Egg Duration and Hatch Rate

Egg masses consisting of 22 to 100 eggs per mass, laid within the previous 24 h, were collected and used in this experiment. Five egg masses were assigned to each of six growth chambers (Model I30VL, Percival Scientific, Perry, IA, USA) programmed to six constant-temperature treatments: 16, 20, 25, 28, 32, and 36 °C at a photoperiod of 14:10 h (L:D). The number of temperature treatments evaluated per life stage varied based on the availability of insects from the laboratory colony. The egg masses were placed individually in cells of 8-cell trays, each cell containing a wet cotton ball to maintain moisture. All growth chambers were operated simultaneously and HOBO^®^ data loggers (ONSET, Bourne, MA, USA) were placed in each chamber to monitor temperature and humidity levels throughout the experiment. Humidity in the growth chamber was maintained at 60 ± 10% RH, though RH in individual cells may have been higher. The number of days until hatching and the number of neonates were recorded daily. The experiment was repeated twice, and the cumulative totals at the end of the experiment were used for data analysis.

#### 2.2.2. Duration of Larval and Pupal Stages

In this experiment, one neonate was placed in each 30 mL cup containing approximately 20 mL of artificial diet. A total of 60 neonates were assigned to each temperature treatment. The constant-temperature treatments were: 16, 18, 20, 22, 25, 26, 28, 30, 32, 34, and 36 °C. Similar to the egg duration and hatch rate experiments, growth chambers were operated simultaneously, and temperature and humidity were monitored throughout the experiment. The larvae were checked every two days, and their diet was replaced every five days to maintain optimal conditions. The number of days to pupation (larval duration) was recorded for each larva. Upon pupation, each pupa was weighed and identified as male or female. The number of days until adult emergence (pupal duration) was also recorded. This experiment was conducted twice, and the pooled cumulative data were used for analysis.

#### 2.2.3. Oviposition and Adult Longevity

Pupae were sorted by sex [11] and newly emerged male and female moths were paired and placed in paper cups containing an oviposition substrate (rolled germination paper) and a 1:1 honey-water solution. Seven pairs were assigned to each of the five temperature treatments: 18, 22, 26, 30, and 34 °C. Growth chambers were operated simultaneously, and temperature and humidity were monitored. The oviposition substrates were checked daily for eggs and replaced each time. The total number of eggs laid per female was recorded, along with the lifespan of adults in days.

### 2.3. Thermal Tolerance Under Short- and Long-Day Photoperiods

*Eoreuma loftini* neonates, within 24 h of hatching, were collected from the colony and transferred to cups containing diet and placed in growth chambers either in a short photoperiod (10:14) or in a long photoperiod (14:10) L:D conditions at 25 °C. When larvae reached fourth instars, they were collected, starved, weighed, and transferred to cups containing fresh artificial diet [10]. Head capsule width was also also measured using a digital caliper (0–200 mm, SKU: 01,407 Neiko, Herten, Germany). The collected larvae from each photoperiod were then acclimated to either 18 °C or 30 °C for 24 h to reduce temperature shock from exposure to low or high temperature extremes, respectively. The larvae acclimated at 18 °C were then exposed to either 0 °C or −5 °C, while those acclimated at 30 °C were exposed to either 40 °C or 45 °C. Each day for five days, 10 larvae (at 0 and 40 °C) and 15 larvae (at −5 °C and 40 °C) were taken out and allowed to recover at room temperature (26 °C) for 24 h. Mortality was assessed after the recovery period. The larva was considered dead if there was a lack of movement after stimulation with a paint brush or forceps.

### 2.4. Data Analysis

Data from the oviposition and larval duration data were analyzed using univariate general linear models (PROC GLM; SAS Enterprise 9.4, SAS Institute, Cary, NC, USA) with temperature as the main factor. The covariate ‘sex (male or female)’ was initially included in the model but removed when found to have no effect. Pupal duration and adult longevity data were analyzed using univariate GLM with temperature, sex, and their interactions as main factors. Egg duration, hatch rate, oviposition rate, and egg viability data were analyzed using univariate GLM with temperature as the main effect. Data residuals were plotted after each analysis and were found to be normal and homogenous. Tukey post-hoc tests (α = 0.05) were performed for all significant main factor effects.

To quantify the development rate and degree-day requirements for *E. loftini*, the mean development time was first calculated for each temperature. The development rate was derived as the reciprocal of mean development time (1/Days). Linear relationships (egg, larvae, pupae, adult, and immature stages) between temperature and development rate [*R(T) = a + bT*], where *T* is temperature, and *a* and *b* were estimates of intercept and slope, respectively, were modeled using least squares linear regression (PROC REG). Degree days for each stage were calculated as the reciprocal of the slope *b* (DD = 1/*b*). The base temperature threshold was estimated by the intersection of the regression line at *R(T)* = 0, *T*_0_ = −*a*/*b*.

Mortality data from thermal tolerance experiments were analyzed using logistic regression (PROC PROBIT) with photoperiod and time as the main effects. Larval weight and head capsule data were analyzed using univariate GLM with photoperiod as the main factor. All analyses were performed using SAS version 9.4 (SAS Institute, Cary, NC, USA).

## 3. Results

### 3.1. Eoreuma loftini Development Under Varying Temperature

**Figure 1:**
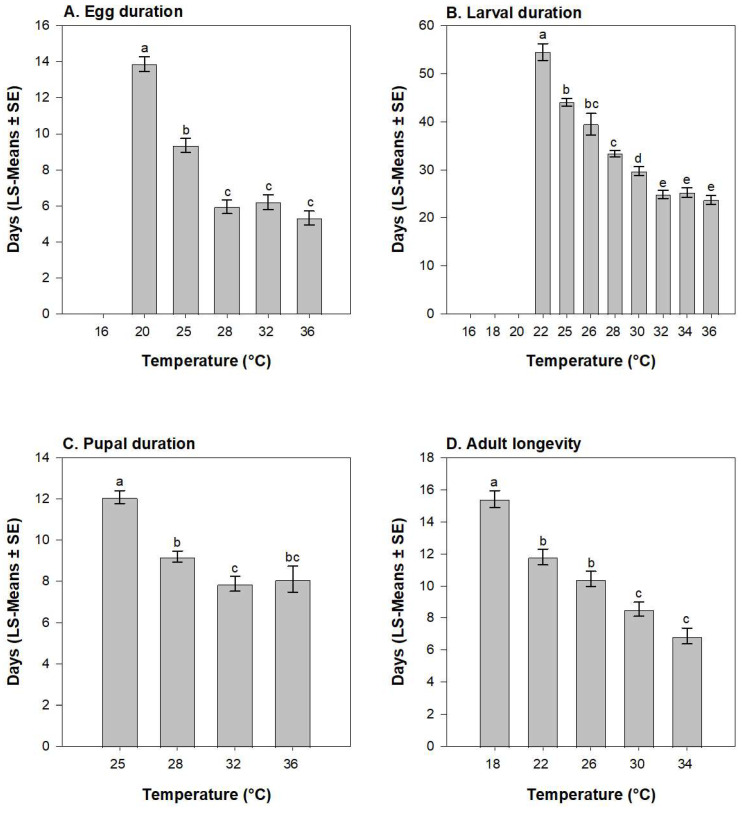
Influence of temperature on duration of *E. loftini* egg stage (**A**), larval development (**B**), pupal stage (**C**), and adult longevity (**D**). Bars within a figure which share a letter are not significantly different (Tukey’s HSD, α = 0.05).

The duration of the egg stage was influenced by temperature (*F* = 78.15, df = 4, 43; *p* < 0.001) with the longest duration at 20 °C and shortest at 28–36 °C (Figure 1A). Eggs did not complete development at temperatures < 20 °C. Larval duration (days to pupation) was influenced by temperature (*F* = 4.56; df = 4, 37; *p* = 0.004) and was approximately 2-fold greater at 22 °C than at 32–36 °C (Figure 1B). Larvae did not complete development below 22 °C. Pupal duration was influenced by temperature (*F* = 4.56; df = 4, 37; *p* = 0.004), but not sex or the temperature × sex interaction. Pupal duration was greatest at 25 °C and decreased with increasing temperature (Figure 1C). Adult longevity was influenced by temperature (*F* = 18.94; df = 9, 74; *p* < 0.001), but not by sex or the temperature × sex interaction. Adult longevity was 2.3-fold greater at 18 °C than at 34 °C (Figure 1D).

**Figure 2:**
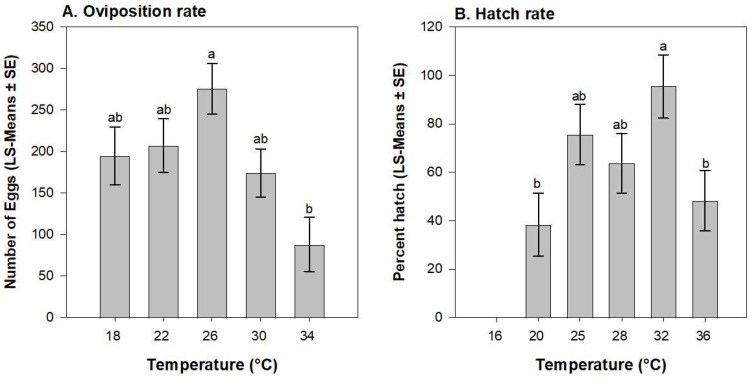
Influence of temperature on *E. loftini* reproductive parameters: (**A**) number of eggs laid per female and (**B**) percentage of egg viability. Bars within a figure which share a letter are not significantly different (Tukey’s HSD, α = 0.05).

The oviposition rate (number of eggs per female) was influenced by temperature (*F* = 4.56; df = 4, 37; *p* = 0.004) with the greatest oviposition occurring at 26 °C and the least at 34 °C (Figure 2A). Egg viability was influenced by temperature (*F* = 3.05; df = 4, 37; *p* = 0.027) with the percentage of hatch success at 32 °C being 2.7-fold greater than at 20 °C (Figure 2B). Eggs were not viable at 16 °C.

The linear model accounted for most of the variability in developmental rates at various temperatures, with a high coefficient of determination (R^2^ ranging from 0.70 to 0.98) (Table 1). The lower developmental threshold ranged from 3.56 to 11.94 °C for the different stages, and the total degree days required for immature development was 909.01.

### 3.2. Thermal Tolerance Under Short- and Long-Day Photoperiod

**Figure 3:**
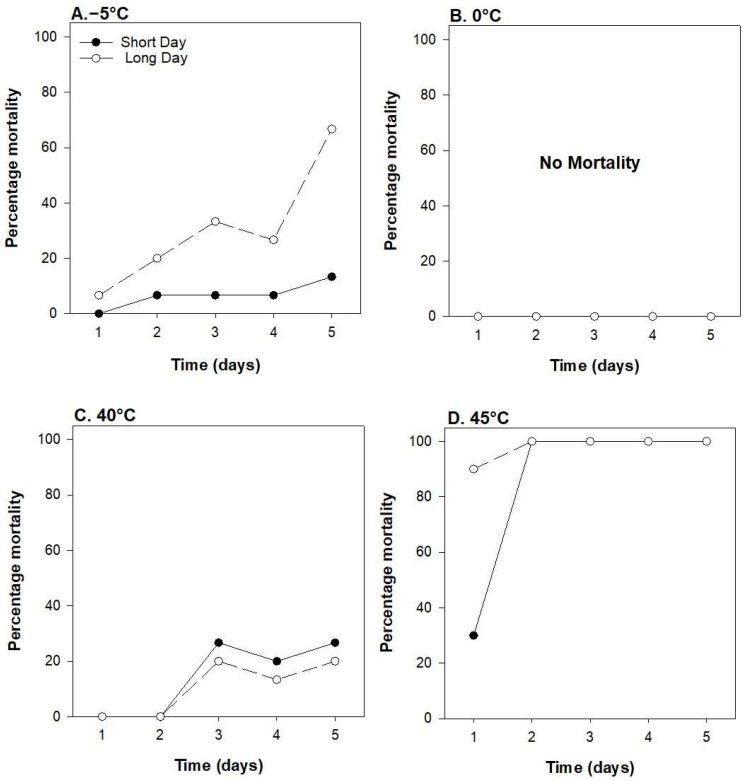
Larval mortality as influenced by photoperiod and time at −5 (**A**), 0 (**B**), 40 (**C**), and 45 °C (**D**).

All larvae survived exposure at 0 °C (Figure 3A). Higher mortality was observed at −5 °C in larvae grown under a long-day photoperiod compared to a short-day photoperiod (χ^2^ = 13.06; *p* < 0.001) (Figure 3B). Time also significantly affected mortality at −5 °C, with a longer exposure time leading to increased mortality (χ^2^ = 11.41; *p* < 0.001). At 40 °C, larval mortality increased with longer exposure time (χ^2^ = 9.46; *p* = 0.002), reaching 20–25% mortality after 5 days (Figure 3C). Mortality at 40 °C was not influenced by photoperiod (χ^2^ = 0.58; *p* = 0.443). All larvae died from the second day onwards at 45 °C (photoperiod: χ^2^ = 0.00; *p* = 0.999, and time: χ^2^ = 0.731; *p* = 0.007) (Figure 3D). Interactions between day and photoperiods were not detected for any temperature (*p* > 0.05).

**Figure 4:**
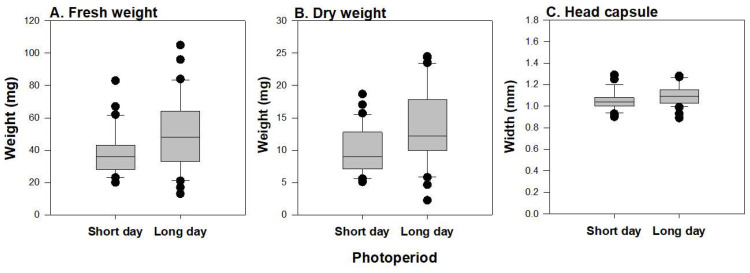
Box plots showing the influence of photoperiod on *E. loftini* larval fresh weight (**A**), dry weight (**B**) and head capsule width (**C**).

Larval fresh weight at the fourth instar was 31% greater under long-day compared to short-day photoperiods (*F* = 12.34; df = 1, 118; *p* < 0.001) (Figure 4A). Similarly, larval dry weight was 38% greater at long-day than at short-day photoperiods (*F* = 17.29; df = 1, 120; *p* < 0.001) (Figure 4B). Head capsule width was influenced by photoperiod (*F* = 7.60; df = 1, 120; *p* = 0.007). Head capsule width was 4% greater under long-day photoperiod than short-day photoperiod (Figure 4C).

## 4. Discussion

This study provides the most comprehensive examination to date of the influence of temperature on *E. loftini* life table parameters and is the first to examine the influence of photoperiod together with temperature regimes. Our findings contribute to the extensive literature on the impact of temperature on insect development while offering new insights into *E. loftini* biology in its invaded range. These results have implications for *E. loftini* ecology in both its native and invaded ranges and are relevant to broader investigations of insect thermal tolerance in the context of climate change and biological invasions [12,13].

The shorter durations of egg, larval, and pupal stages at higher temperatures reported for *E. loftini* herein are consistent with numerous other studies documenting increased developmental rates at increasing temperatures [14,15,16]. The minimal differences in developmental rates at temperatures from 28 to 36 °C reported herein are consistent with previous studies showing that a plateau in development rates above 26 °C occurs in most insects [17]. Similarly, our findings generally agree with previous studies examining *E. loftini* development on artificial diet under varying temperatures [18]. That study reported the shortest larval duration at 32 °C, but we found it to be 21 days, compared to 24 days, in our study. Our study demonstrates that developmental rates do not continue to increase at temperatures > 32 °C. The pupal duration at 32 °C in our study was 7.9 days, slightly longer than the 7.1 days reported by van Leerdam [18]. These discrepancies may be due to the differences in artificial diets used between the two studies. Our developmental durations are slightly shorter than those observed in the van Leerdam study [18] on sugarcane stalk pieces. Thus, artificial diet appears to be nutritionally superior to sugarcane stalks for larval development. The incorporation of sugarcane leaf sheath tissue into artificial diets prolongs larval development and reduces larval weight compared to artificial diets alone [19]. Nutritional factors may have also contributed to the lower oviposition rates reported herein relative to those reported by van Leerdam [18]. However, the effects of nutritional factors cannot be distinguished from inherent differences in reproductive biology which may exist between the two different populations used in our study and that of van Leerdam [18].

Our study sheds new light on *E. loftini* thermal tolerance, which has not been examined in more than 30 years. Although our results are largely in agreement with previous studies conducted by van Leerdam [18] and Rodriguez-del-Bosque [9], a few notable exceptions were observed, suggesting that the influence of temperature on *E. loftini* development may have changed during the course of the species range expansion. The laboratory colony of *E. loftini* used by van Leerdam [18] was established from larvae collected from sugarcane in the Rio Grande Valley of Texas in the early 1980s. Insects in the Rodriguez-del-Bosque study [9] were collected from corn fields in northern Mexico approximately 20 km south of the Rio Grande Valley. The insects used in the present study were from a laboratory colony established from larvae collected from rice in Southern Louisiana from 2018 to 2020. Thus, the two populations of insects are separated by approximately 40 years and 1000 km. The more severe winter freezes in the newly invaded range may have exerted selection pressure, leading to the evolution of improved cold tolerance in *E. loftini* during its range expansion.

Our findings suggest that invasive populations of *E. loftini* have developed increased cold tolerance over time. Rodriguez-del-Bosque [9] reported 60% mortality at 24 h under −5 °C, approximately 6-fold greater than the 10% mortality of long-day larvae exposed to the same temperatures. The exposure of larvae to a winter photoperiod (short-day) decreased mortality at −5 °C to zero at 24 h. Our study did not examine temperatures colder than −5, so it was not possible to determine if survival under −10 and −15 °C, which saw 100% larval mortality in Rodriguez-del-Bosque’s study [9], had similarly increased in Louisiana. Future studies should re-examine the cold tolerance of modern *E. loftini* populations from the same region for more direct comparisons. The relatively minimal mortality of short-day photoperiod larvae up to 5 d at −5 °C suggests that winter mortality is likely minimal throughout much of the species’ current invaded range where temperatures rarely persist below freezing for more than 2–3 days. Survival at −10 °C would provide a better discriminating temperature to help determine the northern extent of its potential invaded range. We found that *E. loftini* larvae survived at 40 °C but experienced complete mortality at 45 °C, consistent with other studies documenting upper thermal limits between 40 and 45 °C for lepidopteran larvae [20,21,22,23]. This thermal tolerance is higher than that of other insects, which suffer substantial mortality at temperatures < 40 °C [24,25].

The improved cold tolerance under a short-day photoperiod reported herein is consistent with previous studies examining photoperiodism in insects [26]. Several studies have demonstrated that shortening the photoperiod leads to reduced super-cooling temperatures [27,28,29]. Shortening the photoperiod often results in the onset of diapause, though this is not required for enhanced cold tolerance [30]. The importance of diapause in *E. loftini* overwintering is unclear [31], though some studies have suggested that as many as 30% of larvae are in a dormant state during winter in subtropical regions of Texas [32]. Studies of *E. loftini* overwintering in more temperate regions of its invaded range are scarcer, though Beuzelin et al. observed numerous larvae infesting non-crop grasses in eastern Texas throughout the winter and did not report a state of dormancy [33]. Wilson et al. [4] reported that *E. loftini* adults were captured in pheromone traps throughout the year in Louisiana, though lower densities were observed in winter months. This suggests that a photoperiod-regulated state of diapause is not prevalent in U.S. populations of *E. loftini*. However, physiological responses to photoperiod have been shown to change in populations of invasive insects undergoing northward range expansion [34]. Thus, inferences from studies in southern Texas may not be applicable to a putatively novel overwintering behavior of *E. loftini* in Louisiana.

The lack of influence of photoperiod on tolerance to upper temperature extremes reported herein contrasts with some studies that have shown increased heat tolerance in insects exposed to increasing photoperiods [12,35,36]. However, this response is less consistent than the influence of a short photoperiod on cold tolerance as other studies have found no effect. Manenti et al. [36] showed increased heat tolerance following increased photoperiod in two out of four species of *Drosophila*.

Both our findings for *E. loftini* cold- and heat-tolerance may not be directly applicable to field populations, however, as our assays used constant temperatures rather than fluctuating regimes which occur in nature. Fluctuating regimes may more accurately assess thermoregulation in insects [16]. Future studies should examine *E. loftini* thermal tolerance under natural temperature fluctuations with adequate acclimation periods, investigate additional parameters such as supercooling point and critical thermal minima [37], and the genetic changes of populations at the northern edge of the invasion.

## Figures and Tables

**Table 1:** Linear regression parameter estimates between temperature and developmental rates (1/D) of *E. loftini* stages.

Stage	Intercept	Slope	R^2^	*n*	Threshold (°C)	Degree Days ^a^
Egg	−0.0660	0.0073	0.88	5	9.04	136.99
Larvae	−0.0215	0.0018	0.96	8	11.94	555.56
Pupae	−0.0114	0.0032	0.70	4	3.56	312.5
Adult	−0.0243	0.0048	0.98	5	5.08	200.00
Immature	−0.0097	0.0011	0.91	4	8.82	909.01

^a^ Total degree-day to complete development.

## Data Availability

Data can be made available upon request to the corresponding author.
